# Production diversification, dietary diversity and consumption seasonality: panel data evidence from Nigeria

**DOI:** 10.1186/s12889-018-5887-6

**Published:** 2018-08-08

**Authors:** Habtamu Yesigat Ayenew, Sibhatu Biadigilign, Lena Schickramm, Getachew Abate-Kassa, Johannes Sauer

**Affiliations:** 1 Agricultural Production and Resource Economics, Technical University Munich, Freising, Germany; 2 Public Health Consultant Ethiopia, Addis Ababa, Ethiopia

**Keywords:** Dietary diversity, Production diversification, Poverty, Seasonality

## Abstract

**Background:**

Despite some improvements towards reducing hunger, malnutrition remains to be a crucial challenge in the developing world. The objective of this paper is to analyze the interplay between production diversity and dietary diversity across different seasons in rural Nigeria. The paper also investigates the relationship across different income quantiles.

**Method:**

The study uses the Living Standards Measurement Study – Integrated Surveys on Agriculture (LSMS-ISA) dataset of the World Bank. We use two rounds of survey data (2010 and 2012) from Nigeria. Data were collected in two visits: at post-planting (from September to November), and at post-harvesting (from February to April). We analyze the relationship between production diversity and dietary diversity using different panel data regression tools.

**Result:**

In post-harvest season, an increase in farm production diversification is associated with an increase with dietary diversity. On the other hand, production diversification does not have a significant contribution to the dietary diversity at post-planting. The analysis reveals that production diversification leads to better diet diversity for households in the second and third income quantiles.

**Conclusion:**

Seasonal variation on the contribution of production diversification on dietary diversity in rural Nigeria calls for the role of seasonally targeted policies. A higher propensity of households in the poorest quantile for malnutrition irrespective of the season suggests the need for targeted and continuous public health and nutrition interventions.

## Background

Despite some improvements towards reducing hunger in the last few decades, malnutrition remains to be a crucial challenge in the developing world [1–4]. Globally, chronically undernourished people are estimated to have reached about 815 million in 2015. About 38 million people are severely food insecure in northern Nigeria, Somalia, South Sudan and Yemen. In those countries, 1.796 million and 4.960 million under five children respectively have severe acute malnutrition (SAM) and moderate acute malnutrition (MAM) [5]. As a response, the 2030 Agenda for Sustainable Development signify and focus on achieving a world without hunger and malnutrition by 2030. In particular, the SDG 2 give a special emphasis to “end hunger, achieve food security and improved nutrition and promote sustainable agriculture by 2030” [6].

Sustainable agriculture captures a special attention in the SDG 2 with the aim to contribute to reduce malnutrition. In addition to the role of agriculture as a source of food, it is a major source of livelihood for the rural poor in the developing world [7–9]. Increased intensification of agriculture and productivity growth can contribute to improve food security and reduction of poverty [8, 10, 11]. On the other hand, increased intensification of agriculture may sometimes lead to decline in food quality [12, 13]. For instance, despite productivity gains from the conversion of pulse land into intensified rice farms with Green Revolution, such a production shift often leads to decline in diet diversity [13]. Similarly, reliance on too much of starchy foods and inadequate nutrient rich diet cause micro-nutrient deficiencies and hidden hunger [14, 15]. As a response to these challenges, a variety of targeted nutrition interventions including nutrient supplementation, promoting dietary diversity, nutrition education and food fortifications have been implemented in many countries to improve the quality of diets [12, 16–18].

Agriculture can be linked to food security and improved nutritional status in multiple and bi-directional pathways. First, agriculture and food production can directly contribute to the nutrition condition of the rural poor. Second, agricultural productivity gain can improve farm income, and income gain in turn can improve nutrition condition. Third, the types and quality of the food items produced in the farm can enhance nutritional quality. A good example could be the effect of farm production diversification on the dietary diversity and nutritional quality.

Understanding the interplay between farm production diversification and dietary diversity is especially relevant in smallholder agriculture and in sub-Saharan Africa (SSA). First, SSA has a hunger prevalence of over 30% and it is a region where the number of malnourished people is increasing with time [19–21]. Second, smallholder subsistence farm production predominates in SSA and own production constitutes the largest proportion of the family diet [22, 23]. Jones, Shrinvas [24] and Sibhatu, Krishna [25] for instance, using data from selected developing counties, show that farm diversification significantly influences the dietary diversity of farming families. A key aspect of this relationship could be the seasonality of agricultural production and its effect on consumption seasonality. Nonetheless, the existing empirical evidence overlooks the relationship across seasons [24, 25]. In addition, the existing literature on the relationship between farm production and consumption did not explore the effect across different income levels.

Vulnerability to hunger can be seasonal in character [26–28]. Smallholder subsistence farmers are often vulnerable to seasonal consumption poverty, and struggle to attain adequate diet for most parts of the year. If this is the case in rural Nigeria, there could be variation on the exposure to malnutrition across seasons. Seasonal consumption poverty and hunger can partly be due to the seasonality of most agricultural products. This is crucial in rural areas of SSA, where production services, storage and market infrastructures are often less developed [29, 30]. As a response, adjusting production and consumption schedules to meet the dietary requirement across different seasons remains to be an important concern in smallholder agriculture [28]. The effect can also depend on the capability and level of poverty of the household. Poor rural households often find it difficult to maintain diverse diet.

It is of special interest in this paper to explore the interplay between farm production diversity, dietary diversity and seasonality using an unbalanced panel data from Nigeria. In this paper, we analyze the implication of farm production diversity on the dietary diversity of the household in two seasons. We separately do the analysis in post-harvest and post planting seasons, and across different income quantiles.

## Methods

### Measurement of dietary diversity and farm diversification

There are multiple approaches to quantify the level of farm diversification (and sometimes biodiversity) and dietary diversity. The following section illustrates the approaches that are used mostly for the calculation of farm production diversification and dietary diversity of households in the sample.

Dietary diversity is often used as an indicator of the food quality, and is constructed from the sum of unique foodstuffs consumed in a specified period of time [31]. There are a number of approaches to measure dietary diversity. The selection mostly depends on the type of foodstuffs used to construct the index, the level of aggregation, the time period used for the construction of diversity index, etc. For instance, the household dietary diversity score (HDDS) is calculated using a 24 h consumption of 113 food types [32]. The household food consumption score (HFCS) on the other hand uses a 7 days food balance sheet of households for the calculation of the index.

There are also variety of approaches based on the level of aggregation in the calculation of the index [24, 33]. The simple food variety count index, for instance, is calculated as the sum of the simple count of all the food items consumed by the household. The household dietary diversity score (HDDS) on the other hand is measured as the sum of the various food groups in the food balance sheet of the households in a specific period.

In this paper, we employ the household dietary diversity score (HDDS) as it better captures if the diet is composed of various dimensions of micro-nutrients. For instance, while wheat or barley count as two different types of foods in the food variety count index, they belong to the cereal category in the food diversity index. As the dataset lacks information on the 24 h format, we use the 7 day food balance sheet for the calculation of the dietary diversity of the household. Accordingly, the household dietary diversity score is calculated from the consumption of 12 food categories (cereals, roots and tubers, pulses, oil and fats, vegetables, fruits, meat, eggs, milk and milk products, sugar and sweets, beverages and alcohol) consumed in a period of 1 week. The index has a continuous score that ranges from 1 to 12. This information is collected twice, at post-harvest and post-planting. This provides the possibility to evaluate the seasonality of dietary diversity of the household. It is often the case to prepare meal together and share within the family in most SSA countries. Furthermore, the food balance sheet data are collected from rural families.

Farm diversification is another important variable for our empirical analysis. Nutrition and development literature measures farm diversification in a number of ways based on the availability of the foodstuff, the required level of aggregation, the purpose of the index, presence of data, etc. [24, 25, 33, 34]. Biodiversity index and aggregated food production category index are commonly applied in empirical studies. The former is a simple count of all unique outputs of crop and livestock activities in the farm [35, 36]. The aggregate food production index measures the sum of the food categories produced by the farm household. We employ the latter for the analysis as the aggregate farm diversification index is superior in measuring the uniqueness of the contribution of a specific output to dietary diversity. For the aggregated food production index, we use nine unique food groups (cereals, roots and tubers, pulses, oil and fats, vegetables, fruits, meat, eggs, milk and milk products, sugar and sweets) produced by the farm household.

### Data

This study uses a large an unbalanced panel data from the Living Standards Measurement Study- Integrated Surveys on Agriculture (LSMS-ISA) of the World Bank from Nigeria. We use the two rounds (of the 2010 and 2012 years) for the analysis. Data were collected in two visits: post-harvesting (from February to April), and post-planting (from September to November). This dataset is a rich panel consisting of a wide range of information including household demographic and socio-economic conditions, production and nutrition related aspects etc. together with geographical and environmental characteristics in the area.

### Statistical analysis

We estimate the relationship between production diversification and dietary diversity of households using unbalanced panel data using Random Effects model (RE), Mundlak random effect model (Mundlak RE) and Fixed Effects (FE) model. We estimate the relationship without controls and by controlling for other factors, and across different seasons. We further estimate the relationship using quantile regression. Unless indicated otherwise (e.g. for the case of quantile analysis), discussions and conclusions are based on the estimation results of fixed effects (FE) model.

## Results

Table 1 presents descriptive statistics of the sample households in Nigeria. In total, we use 6089 observations (3063 in the year 2010 and 3026 in the year 2012). The summary statistics shows that households on average produce about four food categories; and consume food from about six categories at planting and harvesting seasons. This implies that a significant proportion of the diet comes from the market.

**Table 1:** Summary statistics

Variable	Description and measurement	Mean	Std. dev.	Min.	Max.
DIET DIV_H	Dietary diversity at harvest (in count)	6.601	1.906	1	11
DIET DIV_P	Dietary diversity at planting (in count)	6.443	1.996	1	11
PRD DIV	Production diversity (in count)	4.248	3.008	1	11
AGE	Household head age (years)	51.51	15.024	18	112
SEX	Household head sex (0 = female, 1 = male)	.878	.328	0	1
EDUC	Education (year completed)	1.454	1.785	0	15
FAMSIZ	Family size (in count)	5.707	3.072	1	31
LAND	Cultivated land (in hectares)	1.941	3.774	0	108.82
LIVEST	Livestock	1.423	58.287	0	4463.64
DIST	Distance to the market (in kilometers)	72.02	40.16	0.28	214.36

Furthermore, the dietary diversity of households varies in magnitude across the harvesting and planting seasons. The dietary diversity is higher at the time of harvest. The sample comprises of a higher percentage of male headed (87.8%) households.

**Fig. 1:**
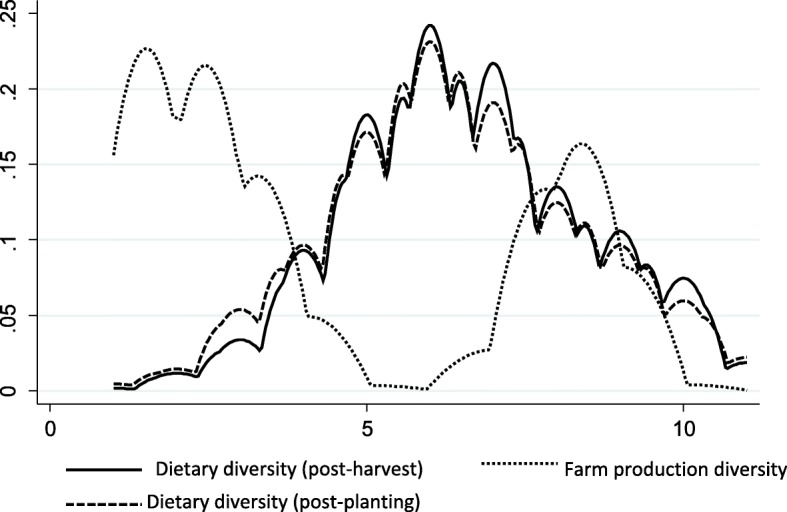
Kernel density estimation of farm diversification and dietary diversity

The kernel density graph (Fig. 1) show that farm production diversity is skewed towards the left and right. This implies that an important proportion of farm households in the sample are engaged in extremely specialized or partially specialized production orientation; and the other substantial proportion is engaged with diversified farm production activities. On the other hand, the pattern of dietary diversity varies from this. The graph indicates that a substantial proportion of rural households consume diverse diet close to the median. In addition, we observe some differences in the pattern of dietary diversity at post-harvest and post-planting. We will explore this relationship in depth in the following section.

Table 2 presents summary statistics of variables across different income quantiles. Compared to households in the middle and higher income quantile, farm households in the first income quantile (the poorest) are older, comprise more female headed households, have lower family size, have low landholding and fewer livestock, and consume less diverse diet. Farm diversification is higher for the poorest compared to those in the second quantile, but lower when it is compared with the rich households. The summary statistics across income quantiles is consistent with the kernel estimation graph, and show the variation across the income quantiles.

**Table 2:** Summary statistics across income quantiles

Variable	1st quantile	2nd quantile	3rd quantile
	Mean (Std. dev.)	Mean (Std. dev.)	Mean (Std. dev.)
DIET DIV_H	6.392 (1.817)	6.392 (1.861)	7.018 (1.971)
DIET DIV_P	6.262 (1.949)	6.304 (1.989)	6.765 (2.012)
PRD DIV	4.074 (2.939)	3.921 (2.957)	4.751 (3.063)
AGE	54.96 (15.965)	49.769 (14.668)	49.787 (14.092)
SEX	.756 (.429)	.912 (.283)	.966 (.183)
EDUC	1.592 (1.942)	1.416 (1.815)	1.355 (1.576)
FAMSIZ	4.769 (2.706)	5.668 (2.836)	6.676 (3.329)
LAND	.776 (2.013)	2.138 (3.467)	2.909 (4.933)
LIVEST	.045 (.087)	.255 (.419)	3.971 (100.94)
DIST	68.472 (38.185)	74.977 (40.787)	72.622 (41.204)
Observations	2030	2030	2029

Table 3 presents estimation results of the relationship between production diversification and dietary diversity using Random Effects (RE), Mundlak Random effects and Fixed Effects (FE) models right after harvesting season (post-harvest). In the table, we report the relationship at post-harvest in a reduced model (column 1, 3, and 5) by including production diversification alone; and the full model (column 2, 4, and 6) by including other control variables.

**Table 3:** Relationship between production diversification and dietary diversity (post-harvest)

Models	RE		Mundlak RE		FE	
PRD DIV	.025^c^ (.006)	.017^b^ (.007)	.025^c^ (.006)	.017^b^ (.007)	.016^b^ (.007)	.019^b^ (.009)
AGE		−.009^c^ (.002)		−.009^c^ (.002)		.003 (.005)
SEX		.209^c^ (.073)		.209^c^ (.073)		.242 (.340)
EDUC		.023^a^ (.013)		.023^a^ (.013)		−.003 (.025)
FAMSIZ		.052^c^ (.008)		.052^c^ (.008)		−.017 (.20)
LAND		−1.3e-07 (5.6e-07)		−1.3e-07 (5.6e-07)		−1.1e-07 (7.9e-07)
LIVEST		2.2e-04 (3.3e-04)		2.2e-04 (3.3e-04)		7.5e-04^a^ (4.3e-04)
DIST		−.005^c^ (7.3e-04)		−.005^c^ (7.3e-04)		
Year	Yes	Yes		Yes		Yes
Region	Yes	Yes		Yes		Yes

Notes: *N* = 6089 ^a^, ^b^ and ^c^ represent significance at 1, 5 and 10% probability levels. Numbers is parenthesis represent standard errors

As shown in Table 3, production diversity significantly and positively influences the dietary diversity of rural households. The elasticity of production diversification is lower when we control unobserved heterogeneity using Mundlak RE and FE models. In estimations without control variables, a unit increase in the farm production diversity increases the dietary diversity with about 0.025 units in RE and Mundlak RE models, and with about 0.016 in FE model. This variation of the effect of production diversification across different estimation methods is smaller when we include control variables (Columns 2, 4 and 6). Based on the results presented in Table 3, we can conclude that, an increase in production diversification leads to a substantial improvement in dietary diversity of the family.

Among the control variables, distance to the nearest market, age of the household head, sex of the household head, family size and education level of the household head do significantly influence the dietary diversity at post-harvest in RE and Mundlak RE models. Nonetheless, most of this relationship does not exist anymore when we do the estimation using FE model. The only variable that appears to be significant is livestock holding, and it contributes positively for dietary diversity.

In Table 4, we report the relationship between production diversification and dietary diversity at post-planting. Similar to the approach we use for the analysis at the time of harvest (Table 3), we first estimate the relationship without controls.

**Table 4:** Relationship between production diversification and dietary diversity (post-planting)

Models	RE		Mundlak RE		FE	
PRD DIV	.015^a^ (.007)	.005 (.007)	.009 (.008)	.012 (.010)	.009 (.008)	.013 (.010)
AGE		−.009^b^ (.002)		.006 (.006)		.006 (.006)
SEX		.195^b^ (.080)		.317 (.373)		−.317 (.374)
EDUC		.023 (.014)		−.014 (.027)		−.014 (.027)
FAMSIZ		.065^b^ (.009)		−.013 (.022)		−.013 (.022)
LAND		1.7e-06^b^ (6.1e-07)		2.4e-06^b^ (8.6e-07)		2.4e-06^b^ (8.7e-07)
LIVEST		2.2e-04 (3.3e-04)		4.5e-04 (4.7e-04)		4.5e-04 (4.7e-04)
DIST		−.005^b^ (7.3e-04)		−.006^b^ (8.1e-04)		
Year	Yes	Yes		Yes		
Region	Yes	Yes		Yes		

Notes: *N* = 6092. ^a^ and ^b^ represent significance at 5 and 10% probability levels. Numbers is parenthesis represent standard errors

The relationship between production diversification and dietary diversity at post-planting is significant only when we estimate them without controls in RE model (Table 4, Column 1). When we control unobserved heterogeneity in Mundlak RE and FE models, and when we control for control variables (Column 2 up to 6), the relationship between production diversification and dietary diversity is not statistically significant. This implies that production diversification has no statistically significant effect on the dietary diversity of households at post-planting in rural Nigeria.

Based on the findings present on Tables 3 and 4, we can conclude two things. First, at post-harvest, production diversity contributes to dietary diversity. Second, the effect of production diversity on dietary diversity varies across seasons, and the effect disappears when the household get closer to the time of post-planting. This finding indicates the decline in the diversity of foodstuffs that people consume at lean periods in Nigeria. This seasonal variation of the effect of production diversification can be attributed to the features of agriculture and related infrastructures in rural areas of the developing world. First, agricultural products are seasonal and perishable. With poor storage facilities in the rural areas of the developing world, there is little possibility to maintain the same level of dietary diversity in off-seasons. Second, smallholder farmers in the developing world often do supply their “marketable surplus” to the market right after the time of harvest. This market transactions right after the time of harvest might have an implication on the dietary diversity of smallholder farmers in rural Nigeria. The decline in dietary diversity in the off-season (post-planting) may imply the possible existence of seasonal consumption poverty in subsistence agriculture. Overall, this indicates the seasonality of hunger and malnutrition in rural Nigeria which calls for seasonally targeted public health and nutrition interventions.

Among the control variables, cultivated land is associated with improved dietary diversity in planting season when we estimate the relationship using RE, Mundlak RE and FE models. However, the magnitude of the effect is very small and it is vital to note that this effect is not there at harvesting. This can be associated with the role of the scale of operation of farming for overall livelihood of the family, and consumption smoothing in the household. With little variation in the magnitude in RE and Mundlak RE models, dietary diversity is likely to be higher when the household resides close to the market. Age and sex of the household head, family size, cultivated land, and market distance are significant determinants of dietary diversity in RE model. Only cultivated land remains to be statistically significant in a FE model. The result implies that most of the control variables are time invariant (or vary with constant value), and the effect will be absorbed by the parameter that we use to control the unobserved fixed effect.

Estimation of the relationship between farm production diversity and dietary diversity across income quantiles reveal the following results (see Table 5). Production diversification has no effect at post-planting, and this is consistent with the result presented in Table 4. At post-harvest, production diversification has no effect for the very poor households in the first income quantile. On the other hand, an increase in production diversification leads to better diet diversity for households in the second and third income quantile. The result implies that poor farmers are worse-off in terms of diet diversity regardless of the season.

**Table 5:** Determinants of dietary diversity across income quantiles

	post-harvest			post-planting		
	1st quantile	2nd quantile	3rd quantile	1st quantile	2nd quantile	3rd quantile
PRD DIV	−.005 (.021)	.044^a^ (.026)	.087^c^ (.022)	−.019 (.023)	.017 (.028)	.041 (.026)
AGE	.019 (.012)	−.015 (.016)	−.021^a^ (.011)	.019 (.013)	.004 (.017)	.002 (.013)
SEX	−.689 (.478)	1.144 (1.961)	−.704 (1.93)	−.319 (.527)	−.902 (2.13)	.103 (2.22)
EDUC	−.034 (.049)	−.056 (.067)	.012 (.067)	.004 (.054)	−.007 (.073)	.079 (.076)
FAMSIZ	.057 (.055)	−.077 (.055)	−.063 (.046)	.012 (.061)	.003 (.059)	−.126^b^ (.053)
LAND	−9.9e-07 (3.9e-06)	−1.5e-07 (2.2e-06)	−1.1e-06 (1.1e-06)	4.6e-06 (4.3e-06)	5.0e-06^b^ (2.4e-06)	7.2e-07 (1.3e-06)
LIVEST	−.486 (.789)	.334 (.248)	−.003 (.002)	.719 (.869)	−.131 (.269)	.001 (.003)
DIST						
Year	Yes	Yes	Yes	Yes	Yes	Yes
Model	Fixed Effect	Fixed Effect	Fixed Effect	Fixed Effect	Fixed Effect	Fixed Effect

Notes: *N* = 6092. ^a^, ^b^ and ^c^ represent significance at 1, 5 and 10% probability levels. Numbers is parenthesis represent standard errors

## Discussions

Despite some improvements on food security situation in the last couple of decades, hunger and malnutrition remain crucial challenges in SSA [26, 30, 37]. Among others, the introduction of production and productivity enhancing technologies do contribute to this improvement. However, researchers also report some concerns in this regard. Masanjala [37] in a study in Malawi show that productivity gains in cash crops might not bring significant improvements in per capita food intake. The introduction of productivity enhancing technologies brought a great deal of interest to convert diversified land use systems to specialized farm. This shifts in to specialization can have detrimental impact on dietary quality [2, 3]. For example, researchers highlighted the decline in the food quality at the time of green revolution as a result of the conversion of pulse farms to rice production plots [13]. Conversely, diversified farming can contribute for diet diversity and improve nutritional quality [24, 25]. This is particularly relevant for most subsistence farm households as they rely on their farms to meet their dietary requirements [27].

Our empirical analysis shows that farm production diversification of the households contribute to dietary diversity at post-harvest season in Nigeria. The result indicates the importance of own production for diet quality, and reveals the contribution of farm production diversification to improve the quality of diets at harvest season in rural Nigeria. Previous empirical works also document the role of production diversification to improve the dietary diversity of farm households in the developing world [24, 25]. However, the existing empirical evidence did not explicitly show the relationship across different seasons.

Our analysis shows that the relationship between farm production diversification and dietary diversity does not exist at post-planting. This implies the existence of seasonal consumption poverty that is reflected with the decline in diet diversity in rural Nigeria. The seasonality of the effects of production diversification on dietary diversity can be associated with the features of agriculture and related infrastructures in rural areas of the developing world. First, seasonality and perishability of agricultural products and poor storage facilities in rural Nigeria might contribute to this. With poor storage facilities in the rural areas of the developing world, there is little possibility to maintain the same level of nutrition quality and dietary diversity in off-seasons [30, 38, 39]. Smith, Alderman [38] for instance indicate the presence of chronic shortage of vegetable and fruit crops in dry seasons in SSA. Breaking this pattern requires technologies that assure year round supply of agricultural outputs and food including irrigation. As Burney and Naylor [30] show, small-scale irrigation can help to improve nutritional status of farmers by reducing seasonal food production shocks in Benin. This problem also requires a continuous research and innovation to extend the shelf life of some food items, and this in turn can help to maintain diverse diet across seasons in the household. Second, smallholder farmers in the developing world often do supply their “marketable surplus” to the market right after the time of harvest often with lower price. This cheap food supply right after the time of harvest (which often is followed with purchase with high price at planting time) has implications on the dietary diversity. As Byerlee, Jayne [40] and Ellis and Manda [26] show, seasonal price instability in Africa can contribute to vulnerability to hunger. Roba, O’Connor [28] also document the existing seasonal variation in the level of undernutrition for lactating mothers in Ethiopia. In one way or the other, the seasonal nature of agriculture itself, and poor rural infrastructure in Nigeria can contribute to the decline in the dietary diversity and nutrition quality of households at lean season.

Another key issue that we address in this paper is the variation on the effect of farm production diversification on dietary diversity across income quantiles. The quantile estimation at post-harvest reveals that farm diversification has no effect on diet diversity for the poorest quantile. Nonetheless, an increase in production diversification leads to better diet diversity for households in the second and third income quantiles. The analysis indicates that poor farmers in rural Nigeria consume less diverse diet compared to those in the middle and highest income quantiles. This paper also provides an evidence that production diversity has little to do to the improvement of diet diversity for the poorest quantile regardless of the agricultural production season.

This paper augments the works of Jones, Shrinvas [24], Sibhatu, Krishna [25, 41]. This finding is consistent with the existing evidence by showing the contribution of farm production diversity at post-harvest. Unlike these existing empirical evidences, our paper explores the relationship between production diversification and dietary diversity across different seasons, and shows that production diversification contributes positively for dietary diversity only at post-harvest. While existing papers also either explicitly or implicitly assume that the effect is consistent across various income levels, our paper confirm variation of the effect of farm production diversification on dietary diversity across the different income quantiles.

### Limitations of the study

There are some limitations of this study. First, this research is based on weekly consumption data at post-harvest and post-planting. The analysis can only reflect consumption in that specific period, ad might not be representative for the whole season. In addition, this consumption data is based on the household heads’ self-report. Second, we only use two data points (2010 and 2012) for the empirical analysis. Using more time points for the analysis could be more informative. Despite these limitations, this study contributes to the existing literature on production diversification, dietary diversity and seasonality in rural Nigeria.

## Conclusions

Overall, we show that production diversification in the farm can contribute to improve the dietary diversity of the household at post-harvest, and this is not the case at post-planting. In addition, unlike rural households in the second and third quantile, farm production diversification has no effect on diet diversity even at post-harvest for households in the first quantile. This finding provides some key insights for policy that aim to improve dietary diversity and food security in rural Nigeria. First, farm production diversification can play a vital role to improve nutritional quality, and it is vital to focus on nutritionally sensitive food production interventions. This finding can also support policies aimed at increasing the diversity of farm as one key strategy to improve diet quality. Such efforts are particularly relevant especially if targeted towards nutritionally rich crop and livestock products. Second, the result confirms that food availability and diet diversity are seasonal in rural Nigeria. This suggests for the role of seasonally targeted nutrition interventions. In addition, such seasonal decline in dietary diversity calls the need for interventions to improve nutrition and market infrastructures in rural Nigeria. Third, the effect of production diversity on diet diversity varies across different income quantiles, and is not significant for the households in the first income quantiles (the poorest ones). Such a higher propensity of the poor to stay malnourished regardless of the season of food production in rural Nigeria suggests the need for targeted and continuous public health and nutrition interventions.

## Abbreviations

DD: Dietary diversity
FE: Fixed effects
HDDS: Household dietary diversity score
IV: Instrumental variable
LSMS-ISA: Living Standards Measurement Study – Integrated Surveys on Agriculture
PD: Production diversity
RE: Random effects
SSA: Sub-Saharan Africa
