# Correction to: Production diversification, dietary diversity and consumption seasonality: panel data evidence from Nigeria

**DOI:** 10.1186/s12889-018-5991-7

**Published:** 2018-08-29

**Authors:** Habtamu Yesigat Ayenew, Sibhatu Biadgilign, Lena Schickramm, Getachew Abate-Kassa, Johannes Sauer

**Affiliations:** 1 Agricultural Production and Resource Economics, Technical University Munich, Freising, Germany; 2 Public Health Consultant Ethiopia, Addis Ababa, Ethiopia

## Correction

It has been highlighted that the original article [1] contained a typesetting mistake in the name of Sibhatu Biadgilign. This was incorrectly captured as Biadigilign in the original publication which has since been updated.
